# First Attempt To Validate Human IgG Antibody Response to Nterm-34kDa Salivary Peptide as Biomarker for Evaluating Exposure to *Aedes aegypti* Bites

**DOI:** 10.1371/journal.pntd.0001905

**Published:** 2012-11-15

**Authors:** Emmanuel Elanga Ndille, Souleymane Doucoure, Georgia Damien, François Mouchet, Papa Makhtar Drame, Sylvie Cornelie, Herbert Noukpo, Sandra Yamadjako, Armel Djenontin, Nicolas Moiroux, Dorothee Misse, Martin Akogbeto, Vincent Corbel, Marie-Claire Henry, Fabrice Chandre, Thierry Baldet, Franck Remoue

**Affiliations:** 1 Institut de Recherche pour le Développement (IRD), Maladies Infectieuses et Vecteurs, Ecologie, Génétique, Evolution et Contrôle (MIVEGEC), UMR IRD 224 - CNRS 5290 – Universities of Montpellier 1 and 2, Cotonou, Republic of Bénin; 2 Centre de Recherches Entomologiques de Cotonou (CREC), Ministère de la Santé Publique, Cotonou, Republic of Bénin; 3 Institut de Recherche pour le Développement (IRD), MIVEGEC Unit, Montpellier, France; 4 Centre de Coopération Internationale en Recherche Agronomique pour le Développement (CIRAD), CMAEE Unit, Montpellier, France; National Institute of Allergy and Infectious Diseases, United States of America

## Abstract

**Background:**

Much effort is being devoted for developing new indicators to evaluate the human exposure to *Aedes* mosquito bites and the risk of arbovirus transmission. Human antibody (Ab) responses to mosquito salivary components could represent a promising tool for evaluating the human-vector contact.

**Methodology/Principal findings:**

To develop a specific biomarker of human exposure to *Aedes aegypti* bites, we measured IgG Ab response to *Ae. aegypti* Nterm-34 kDa salivary peptide in exposed children in 7 villages of Southern Benin (West Africa). Results showed that specific IgG response presented high inter-individual heterogeneity between villages. IgG response was associated with rainfall and IgG level increased from dry (low exposure) to rainy (high exposure) seasons. These findings indicate that IgG Ab to Nterm-34 kDa salivary peptide may represent a reliable biomarker to detect variation in human exposure to *Ae. aegypti* bites.

**Conclusion/Significance:**

This preliminary study highlights the potential use of Ab response to this salivary peptide for evaluating human exposure to *Ae. aegypti*. This biomarker could represent a new promising tool for assessing the risk of arbovirus transmission and for evaluating the efficacy of vector control interventions.

## Author Summary

*Aedes aegypti* mosquito is the primary vector of major (re)-emerging human arboviruses, such as Dengue and Chikungunya. In absence of effective treatment and vaccine, the evaluation of human exposure to vector bites is crucial to estimate the risk of the viruses' transmission. Currently, exposure to *Aedes aegypti* bites is mainly evaluated by entomological methods which are indirect and fastidious to apply on a large scale. Human antibody (Ab) responses to arthropod salivary proteins were shown as a useful indicator of exposure to arthropod vector bites. Nevertheless, the whole saliva could not be a specific tool because some families of salivary proteins are common between many arthropod vectors. To develop a specific biomarker of exposure to *Aedes aegypti* bites, we assessed the evolution of IgG Ab response to *Ae. aegypti* Nterm-34 kDa salivary peptide in exposed children. The results indicate that children exposed to the bites of *Ae. aegypti* could develop specific Ab response to Nterm-34 kDa salivary peptide. This specific IgG response presented high inter-individual heterogeneity and increased significantly during the *Ae. aegypti* exposure season. Taken together, these preliminary results suggest that Ab responses to Nterm-34 kDa salivary could constitute a relevant immuno-epidemiological indicator for evaluating human exposure to the *Ae. aegypti* vector and by consequence the risk of arbovirus transmission.

## Introduction

Numerous mosquito species of the genus *Aedes* (Dipteria: Culicidae) are vectors of major (re)-emerging human arboviruses, such as Dengue and Chikungunya. *Aedes aegypti* species is the primary vector of these diseases worldwide. No effective treatment and vaccine are currently available and the transmission can only be reduced or interrupted by controlling mosquito populations and by preventing the human-vector contact.

Exposure to *Aedes aegypti* bites is currently evaluated by entomological methods, at immature stage (eg: number of positive breeding habitats) and/or adult stage (collection of adult mosquitoes by traps, Pyrethrum Spray Catch and human landing catches). These methods present several limitations, such as poor capacity to predict epidemics [1] and for addressing the number of adults vectors produced over time [2]. These methods are labor-time consuming and costly regarding large-scale follow up of mosquito density required. Furthermore, larval and pupal indices target immature stages and do not measure the exposure to adult bites. The density of adult females could be closely associated with the disease incidence [3], [4], but adults collection of *Ae. aegypti* females is fastidious and hard work. These current entomological methods are mainly applicable at the community level and cannot be used to gauge the heterogeneity of individual exposure. They are not accurate to assess individual attractiveness to mosquitoes or other environmental and socioeconomic factors which could induce important variations in individual exposure to vector bites. In order to improve vector control and to predict the risk of arboviruses transmission, complementary methods and indicators are urgently need to evaluate the real human exposure to *Ae. aegypti* bites.

One promising approach is to quantify the human antibody (Ab) response to arthropod salivary proteins used as a biomarker of human exposure to mosquito bites [5]. At the time of biting, the vector injects in the host skin, saliva containing bioactive molecules which facilitate blood feeding [6]. Some of these molecules induce specific Ab response in individuals exposed to bites [7]. Previous studies have shown that anti-saliva Ab response could be an useful indicator to measure the human exposure to arthropod vector bites such as ticks [8], *Triatoma* [9], *Phlebotomus* [10], *Glossina* [11] and *Anopheles* species [12]–[14].

Concerning *Aedes* genus, studies on human allergic reactions have suggested that quantitative evaluation of anti-saliva Ab responses could give a measure of human exposure to *Aedes* bites [15], [16] and increased during rainy season [17]. A significant increase in the anti-saliva Ab response was also observed according to seasonal and spatial *Ae. caspius* density in Southeast France [18]. Regarding *Ae. aegypti* species, it has been demonstrated that IgM and IgG responses to whole saliva could be promising indicator of *Ae. aegypti* exposure in temporarily exposed populations [19]. One study in tropical countries showed that IgE and IgG4 responses to *Ae. aegypti* saliva could be detected in young Senegalese children and that their level increased during the rainy season [20]. Interestingly, IgG response to *Ae. aegypti* saliva was positively associated with entomological indicators in a study conducted in urban area in Bolivia [21]. Recently, our team has shown that IgG Ab level to *Ae. albopictus* can evaluate the exposure to this species in adult individuals [22]. This study demonstrated also a low-level immune cross-reactivity between *Ae. albopictus* and *Ae. aegypti* saliva suggesting the potential to develop specific biomarker to each species.

These results established that specific Ab response against arthropod saliva could evaluate human exposure to vectors bites. Nevertheless, the whole saliva could not be used as convenient biomarker because some families of salivary proteins are common to many bloodsucking *Diptera* [6]. This could induce potential cross-reactivity which potentially skew and/or overestimate the evaluation of exposure to a specific vector. In addition, the use of whole saliva presents other drawbacks such as: i) lack of reproducibility between saliva batches and ii) its adequate production needed for large-scale studies. An optimization of this indicator would be the identification of specific proteins and/or peptides. In this way, our team has validated one salivary peptide (gSG6-P1) as pertinent specific biomarker of exposure to *An. gambiae* and *An. funestus* bites [23]–[25]. By immuno-proteomic approach, a recent study had identified 15 proteins in the sialome of female of *Ae. aegypti* to be potentially antigenic [26]. Among them, the putative 34 kDa family secreted salivary protein appeared specific to *Aedes* genus. Using similar approach than used for gSG6-P1 peptide, the N-terminal extremity peptide (Nterm-34 kDa peptide) of the 34 kDa protein appeared to be an interesting candidate for validation as a biomarker specific to *Ae. aegypti* bites.

The present study aimed at determining whether the IgG Ab response to Nterm-34 kDa peptide could be a biomarker of exposure to *Ae. aegypti* bites in African children living in area of exposure to this vector species. The immunological follow up of a cohort of children was carried out for two years and the changes in specific IgG level were evaluated according to the rainfall quantity and the season of exposure.

## Materials and Methods

### Ethics statement

This study followed the ethical principles according to the Helsinki Declaration and was approved by the National Ethical Committee of Benin (IRB 00006860) and the IRD ethical committee (April 2008). Written informed consent was obtained for all children enrolled in the study and signed by one of their parents.

### Studied population

The study was carried out in rural area of the Ouidah-Kpomassé-Tori Bossito (OKT) health district in southern Benin (West Africa). This site is characterized by a sub-equatorial climate with two dry seasons (from December to March and from August to September) and two rainy seasons (from April to July and from October to November). The annual average of rainfall is around 1,200 mm of which 700–800 mm during the major rainy season and 400–500 in the short rainy season. In this area, a previous study indicated that *Ae. aegypti* is the major *Aedes* species caught inside and around the households [27].

Data for the present study were collected during a longitudinal survey conducted between February 2008 and October 2009 in 7 villages of the OKT health district (1 = Aidjédo; 2 = Dokamé; 3 = Kindjitokpa; 4 = Guézohoué; 5 = Hékandji; 6 = Satré; 7 = Wanho). After census, 420 children (60 for each village) aged from 0 to 60 months old were randomly selected as previously described [28]. Children were visited every 6 weeks and overall 14 visits were conducted during the studied period. At each visit, a dried blood spot was collected in filter-paper from each individual for immunological analysis. The immunological assays were performed on a sub-sample (n = 205) of children for whom blood spots were available for, at least 12/14 visits (89 of 205 children missed one [n = 53] or two [n = 36] visits). No newborn during the study period was included in the present study. All filter papers were kept at +4°C before used.

### Nterm-34 kDa salivary peptide

**Figure 1:**
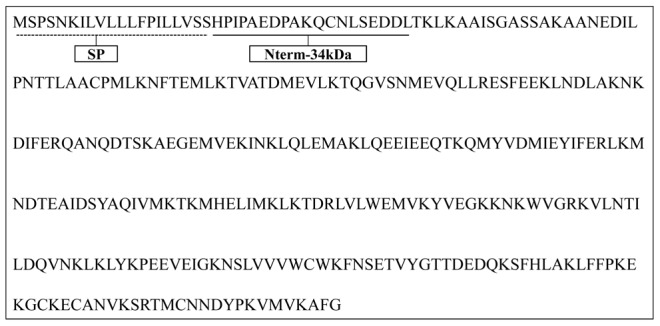
Amino-acid sequence of Nterm-34 kDa Peptide. Amino-acid sequence of the putative 34 kDa family secreted salivary protein of *Aedes aegypti* (gi: 94468336, NCBI database) is presented and sequence of the Nterm-34 kDa peptide is underlined. Signal peptide (SP) sequence is indicating by dotted underline.

As previously described for *An. gambiae* [23], a peptide design strategy using bio-informatic tools was conducted to identify the potential antigenic properties of the Nterm-34 kDa salivary peptide and to select it as candidate biomarker of exposure to *Ae. aegypti* bites. The antigenicity of this N-terminal extremity peptide (19 amino-acids) of the putative 34 kDa family secreted salivary protein (gi|94468336; [29]; figure 1) was computerizing predicted with the BcePred and the FIMM databases. In addition, sequence alignments with the Blast program in Vectorbase database demonstrated the specificity to *Ae. aegypti* by comparison with known genomes and EST libraries of other mosquitoes or organisms. The Nterm-34 kDa peptide was then synthesized, purified (>95%) by Genepep SA (St-Jean de Vedas, France). The peptides were shipped in lyophilized form and then resuspended milliQ water and stored in aliquots at −20°C until their use.

### Evaluation of human IgG Ab levels

Enzyme-linked immunosorbent assay (ELISA) was carried out to evaluate the level of IgG Ab to Nterm-34 kDa peptide in eluates obtained from standardized dried blood spots (1 cm diameter). All ELISA conditions were determined after several preliminary experiments. Samples were eluted by incubation in 350 µl of phosphate buffer (PBS+Tween 0.1%, Sigma-Aldrich, St. Louis, MO) at 4°C for 24 hours. The peptide (10 µg/mL in 100 µl of PBS) was coated at 37°C for 150 minutes onto 64 wells of a 96-well Maxisorp plates (Nunc, Roskilde, Denmark). For each individual sample, one “no antigen” well will be performed to measure the individual non-specific ELISA reactivity by using only 100 µl of PBS for the coating as previously described [23], [25]. Plates were blocked using 300 µl of Protein-Free Blocking-Buffer (Pierce, Thermo Scientific, France) for 45 minutes at 37°C. Each eluate was incubated at 4°C overnight at 1/20 dilution in PBS-Tween 1% in two wells containing peptide and in one “no antigen” well (100 µl for each well). Mouse biotinylated Ab to human IgG (BD Bioscences, San Diego, CA) was incubated at a 1/1000 dilution in PBS-Tween 1% (90 minutes at 37°C) and peroxidase-conjugated streptavidin (GE Healthcare, Orsay, France) was added (1/1000 dilution in PBS-Tween 1%; 60 minutes at 37°C). Colorimetric development was carried out using 2,2′-azino-bis (3-ethylbenzthiazoline 6-sulfonic acid) diammonium (ABTS; Thermo Scientific, France) in 50 mM citrate buffer (pH 4) containing 0.003% H_2_O_2_ and absorbance (OD) was measured at 405 nm. In parallel, specific IgG Ab levels were also evaluated in individuals (n = 10) living in the North of France and with no known exposure to *Ae. aegypti* mosquito and were used to calculate the specific immune response threshold (TR). Individual results were expressed as the ΔOD value calculated according to the formula ΔOD = ODx−ODn, where ODx represented the mean of individual OD values in antigen wells and ODn the OD value in “no antigen” well. A subject was considered as an “immune responder” if his ΔOD was higher than the TR = mean (ΔDO_unexposed_)+3SD = 0.151.

### Statistical analysis

All data were analyzed with GraphPad Prism5 software (San Diego, CA). After verifying that ΔOD values were not normally distributed, the non-parametric tests were used to compare the ΔOD. Mann–Whitney test was used for comparison of Ab levels of two independent groups and the Wilcoxon matched-pairs test was used for comparison of two paired groups. The non-parametric Kruskal–Wallis test was used for comparison of more than two groups. All differences were considered significant at *P*<0.05.

## Results

### IgG response to salivary Nterm-34 kDa peptide in studied population

**Figure 2:**
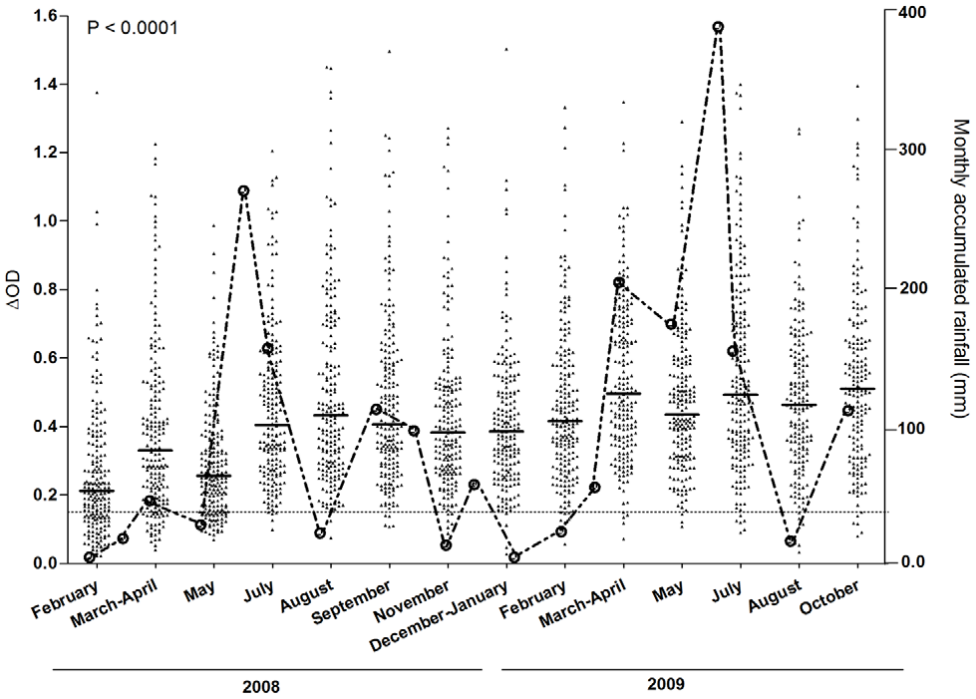
Evolution of IgG antibody response to Nterm-34 kDa peptide and rainfall during the studied period. The evolution of specific IgG level in children and the accumulated rainfall in the studied area are presented for each studied period in 2008 and 2009. Black points indicate individual IgG response (ΔDO) of each child of the studied population. Bars indicate median value in each studied period and dotted line represent the threshold (TR) of specific Ab response (ΔDO>0.151). Statistical significant difference between medians is indicated (non-parametric Kruskal-Wallis test). Rainfalls are presented for each month from February 2008 to October 2009 and were acquired using the GES-DISC Interactive Online Visualization ANd aNalysis Infrastructure (Giovanni) as part of the NASA's Goddard Earth Sciences (GES). Data and Information Services Center (DISC). http://disc2.nascom.nasa.gov/Giovanni/tovas/TRMM).

The evolution of specific IgG level from February 2008 to October 2009 were presented during studied period and compared to the accumulated monthly rainfall registered in the same studied area (Figure 2). For each visit, the median value of IgG Ab level was higher than the specific immune response threshold (TR = 0.151). Specific IgG level was more pronounced i) in July 2008 compared to previous dry season and ii) during all studied period in 2009. High inter-individual heterogeneity in specific IgG Ab level was observed whatever the studied months. The specific IgG response showed significant seasonal variations from the start to the end of the study (P<0.0001, Kruskal–Wallis test). The lowest Ab levels were observed in 2008 during the dry season (from February to May). For both years, IgG level increased significantly from February to March-April (P<0.0001, Wilcoxon matched-pairs test) and from May to July (P<0.0001 and P = 0.002 for 2008 and 2009 respectively, Wilcoxon matched-pairs test), whereas a decrease was observed from March-April to May (P<0.0001, Wilcoxon matched-pairs test). A considerable increase was thereafter observed from July. In contrast, it has been observed a different evolution of IgG level from July to August between 2008 (non significant increase; P = 0.11, Wilcoxon matched-pairs test) and 2009 (significant decrease; P = 0.023, Wilcoxon matched-pairs test). Globally, the specific IgG response was globally higher in 2009 than in 2008.

In the same way, rainfall was more intense during the year 2009 (total = 1, 252.41 mm) than in 2008 (total = 962.23 mm). In 2008, the curve of rainfall was closely associated with the specific IgG response. The rainfall started from February to April, highly increased from May to June, and then decreased from July to August. Interestingly, the peak of rainfall on June 2008 and 2009 was always followed by a peak of IgG Ab level in July.

Similar results were observed for the percentage of immune responders (ΔOD>TR; Table 1). During the first dry season, 63.42% of children were responders (28.78%, 84.84%, 76.74% for February, March-April and May respectively), whereas this percentage reached to an average of 97.28% ([95.95%–98.97%]) from July 2008 to October 2009.

**Table 1:** Characteristics of the studied population during the peak of the dry (February) and the rainy (July) seasons in the years 2008 and 2009.

		Total	Male	Female	Age*(mean-range)	Responders (%)
2008	Dry season	198	108	90	27.57 [04–56]	57 (28.78)
	Rainy season	197	108	89	32.90 [09–60]	195 (98.98)
2009	Dry season	201	109	92	38.90 [16–60]	194 (96.51)
	Rainy season	194	107	87	44.46 [21–60]	188 (96.90)

* The mean age of children and range are expressed in months.

Altogether, these results suggest a possible association between specific IgG Ab response and the intensity of rainfall. This association appeared to be more pronounced in 2008 compared to 2009.

### IgG response to Nterm-34 kDa according to the season of exposure

**Figure 3:**
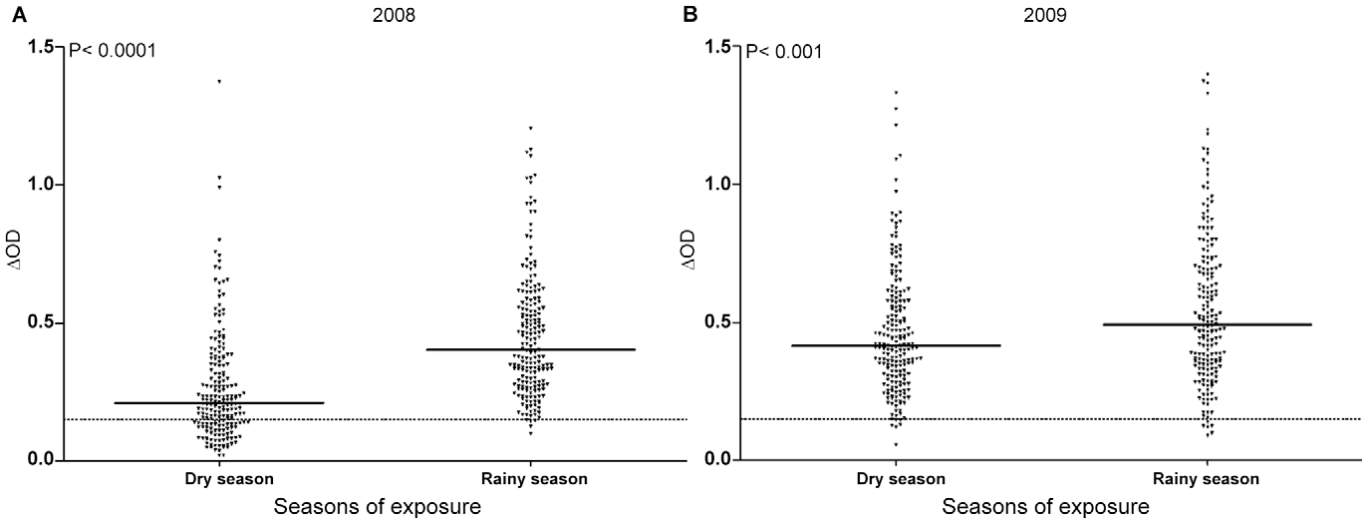
Evolution of individual IgG response to Nterm-34 kDa peptide between dry and rainy seasons. The results are presented for the peak of the dry (February) and the rainy (July) seasons in 2008 (A) and 2009 (B). Black points indicate individual IgG response (ΔDO) and bars indicate the median value for each group. Dotted line represent the threshold (TR) of specific Ab response (ΔDO>0.151) and statistical significant differences between medians are indicated (non- parametric Wilcoxon test).

In the objective to highlight the potential association of specific IgG response with rainfall, IgG Ab level to Nterm-34 kDa peptide was compared between the peak of the dry (February) and rainy (July) seasons in 2008 (Figure 3A) and 2009 (Figure 3B). For both years, specific IgG response increased significantly (P<0.0001 in 2008 and P<0.001 in 2009 Wilcoxon matched-pairs test) in the rainy season compared to dry season. The increase of IgG level was more pronounced in 2008 than 2009. Interestingly, almost all individuals were immune responders (>TR) in rainy season 2008, whereas the median value was closed to TR and only 28.78% of individuals presented positive IgG in dry season. The results of the percentage of immune responders confirmed these differences between 2008 and 2009 (Table 1). High increase was observed from dry season (28.78%) to rainy season (98.98%) in 2008. In 2009, these percentages did not differ between both seasons (96.51% and 96.90%, respectively). These results suggest that the intensity of IgG response to Nterm-34 kDa peptide increased with the rainy season.

### Evolution of IgG Ab response to Nterm-34 kDa according to villages

**Figure 4:**
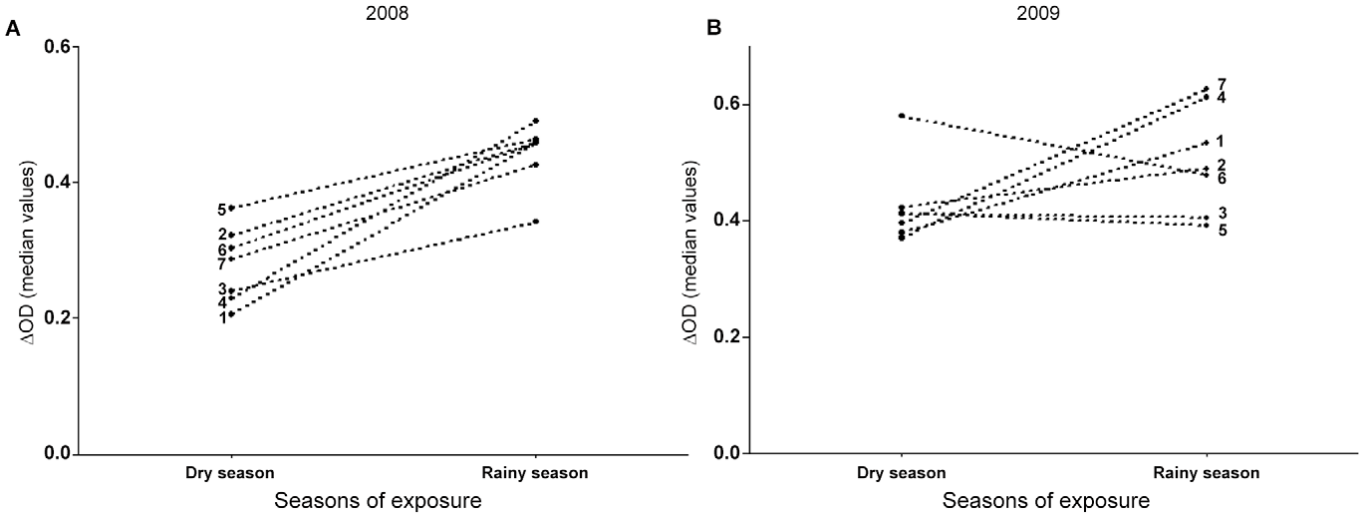
Season-related evolution of IgG response to Nterm-34 kDa peptide according to villages. The evolution of median value of IgG level from the peak of the dry season (February) to the peak of rainy season (July) was presented in 2008 (A) and 2009 (B) according to the 7 studied villages. Numbers from 1 to 7 indicate the villages (1 = Aidjédo; 2 = Dokamé; 3 = Kindjitokpa; 4 = Guézohoué; 5 = Hékandji; 6 = Satré; 7 = Wanho).

The evolution of specific IgG level in the different villages was compared between the peak dry (February) and the rainy (July) seasons in 2008 (Figure 4A) and 2009 (Figure 4B). A significant variation of IgG Ab levels was observed between villages for both years. IgG level significantly increased for all villages from dry to rainy season in 2008 (P<0.0001 for villages 1, 2, 3, 4, 5, 7 and P = 0,003 for village 6, Wilcoxon matched-pairs test) , whereas different trends were observed in 2009. Indeed, increase of IgG Ab levels with the 2009 rainy season was observed only in four villages (1, 4, 7; P<0.0001 and 2, P = 0.89; Wilcoxon matched-pairs test), whereas during the same period, the specific IgG level decreased in village 6 (P>0.05), and appeared to be maintain in villages 3 and 5 (P>0.05; Wilcoxon matched-pairs test).

## Discussion

This study described for the first time, the development of IgG Ab response to *Ae. aegypti* Nterm-34 kDa salivary peptide in human individuals exposed to *Ae. aegypti* bites. The immunological result seemed to confirm the bioinformatic predictions which suggested the potential antigenic properties of this salivary peptide. IgG response to Nterm-34 kDa varied according to the season and was positively associated with the intensity of rainfall. This observation appeared to be more pronounced in 2008 than in 2009. Interestingly, high level of specific IgG response was observed only during rainy season for both years, when almost 100% of children are immune responders. Altogether, these results indicated that the IgG response to Nterm-34 kDa peptide could represent a promising candidate as biomarker of human exposure to *Ae. aegypti* bites.

Evaluation of Ab responses to salivary components might represent an epidemiological marker of *Aedes* exposure. It has been previously reported an increase of Ab response to *Aedes* saliva according to the period of high exposure to mosquito bites [17], [20]. However, the use of whole saliva as biomarker is hampered by its potential to cross-react with others arthropods [30]. To optimizing salivary biomarker for assessing human exposure to *Aedes* bites, this study reports the existence of Ab response to *Aedes* salivary peptide in individuals.

The IgG response to Nterm-34 kDa salivary peptide in children was different between villages and between individuals within the same village. The heterogeneity of IgG level to mosquito saliva components had already been reported by previous studies [20], [23]. It suggests the high heterogeneity of exposure to vector bites among villages and among individuals, as also known for vector-borne diseases transmission. Even if the influence of epidemiological factors (history of exposure, human genetic background, pathogen infections, nutritional status, etc…) on individual Ab response could not be excluded, this result suggests that specific Ab response to Nterm-34 kDa salivary peptide could be pertinent for evaluating the individual exposure to vector bites. This is in accordance with several previous studies showing that such biomarker could be individual indicator for evaluating the real human-vector contact [12], [17], [18], [20], [22], [25]. In addition, variations in the levels of IgG to Nterm-34 kDa peptide appeared related to the intensity of rainfall. The level of specific IgG response globally increased with the increase of rainfall and decreased otherwise. The specific Ab response was higher in 2009 compared to 2008, which was in accordance to the higher rainfall observed in 2009 than in 2008. However, it could be noticed a slight drop of IgG Ab response between August 2008 and February 2009 while rainfall drops considerably. It could probably due to the persistence of considerable exposure to mosquito bites. Indeed, despite the decline of rainfall, it continued to rain during this period, even with weak intensity. It can be favorable for maintaining the proliferation of important densities of *Ae. aegypti* in persistent domestic breeding sites. This could also probably due to the production of mosquitoes in containers filled by people when rain scant as observed in others previous studies [31], [32].

The present results highlight a probable influence of the rainfall on the increase of specific IgG Ab level during the studied period. This association was relevant when the evolution of the specific IgG response was compared between the dry and the rainy seasons for both years, taking into account the peak of the dry (February) and the rainy (July) seasons. Similar influence of rainfall had been previously noticed for Ab response to whole saliva in human populations exposed to *Aedes* [17], [20] and to *Anopheles* bites [12]. Positive association between the levels of IgG Ab response to saliva components and the densities of adult mosquito was clearly reported in several sites and for different mosquito genus; i.e. *Anopheles*, *Aedes* and *Culex* [12], [18], [21], [33], [34]. We can thus hypothesize that the increase of anti-Nterm-34 kDa IgG response during the rainy season could reflect the increase of human exposure to high densities of *Ae. aegypti* mosquito. It is well known that greater proliferation of *Ae. aegypti* adult mosquito occurs during rainfall, especially in African rural context [35]. Additionally, previous investigations indicated that captured female density peaked during times of heavier rainfall in tropical regions [4], [36]. It has been also previously developed a mathematical model which, applied to field data, showed that rainfall triggered the dynamics of *Aedes* mosquito aggressiveness [37]. Collectively, these results indicated that association between rainfall and the level of IgG Ab response to Nterm-34 kDa salivary peptide may reflect the real intensity of human exposure to *Ae. aegypti* bites. Regarding the 2009 season-dependent evolution of specific IgG level according to villages, an increase in the rainy season was observed only for villages 1, 2, 4 and 7. In contrast, the level of specific Ab response appeared unchanged in villages 3 and 5 and decreased in village 6. It could probably indicate that children in villages 3, 5 and 6 could be more protected or less exposed to *Aedes* bites than those in the others village. However, we can't exclude that studied individuals could be exposed to bites of other *Aedes* species such as *Ae. vittatus* which its presence was reported in our study area [27]. Nevertheless, the lack of studies on the sialome of this *Aedes* species has not allowed a bio-informatic comparison with *Ae. aegypti* salivary proteins during the identification of the Nterm-34 kDa peptide.

In this study, the percentages of immune responders were lower and significantly changed at the first three time points in 2008. It could probably be explained by the progressive development of immune response due to cumulative exposure of *Aedes* bites in youngest children. Thereafter, this Ab response level could reach a baseline at determined age and from this age no difference of Ab level can be detected. This hypothesis may explain that the percentage of immune responders remained high and did not differ from July 2008 until the end of study. Nevertheless, in contrast to the proportions of immune responders, the level of specific IgG increased during the rainy season. It suggest, as previously observed for whole saliva [20], that only the level of specific Ab increased during the season of high exposure to *Aedes* bites, but not the percentage of responders.

Altogether, our results showed that individuals exposed to *Aedes* bites could develop IgG response to Nterm-34 kDa salivary peptide. The Ab response differed between individual and increased during season of high exposure to mosquito bites. These data represent a first step to validate the Nterm-34 kDa salivary peptide as a potential biomarker of human exposure to *Aedes* bites. Further studies are needed for final validation taking into account: (i) entomological indicators, even those present considerable limitations; (ii) arbovirus transmission and (iii) others exposed areas with different dynamics of *Aedes* populations. If validated, the level of specific Ab response to Nterm-34 kDa salivary peptide could be used for control and survey programs: (i) to assess the risk of arboviruses transmission and (ii) to evaluate the efficacy of vector control strategies.
